# The Genome Sequence of the Halobacterium salinarum Type Strain Is Closely Related to That of Laboratory Strains NRC-1 and R1

**DOI:** 10.1128/MRA.00429-19

**Published:** 2019-07-11

**Authors:** Friedhelm Pfeiffer, Anita Marchfelder, Bianca Habermann, Mike L. Dyall-Smith

**Affiliations:** a Computational Biology Group, Max Planck Institute of Biochemistry, Martinsried, Germany; b Biology II, Ulm University, Ulm, Germany; c CNRS, IBDM UMR 7288, Computational Biology Group, Aix Marseille Université, Marseille, France; d Veterinary Biosciences, Faculty of Veterinary and Agricultural Sciences, University of Melbourne, Parkville, Australia; Portland State University

## Abstract

High-coverage long-read sequencing of the Halobacterium salinarum type strain (91-R6) revealed a 2.17-Mb chromosome and two large plasmids (148 and 102 kb). Population heterogeneity and long repeats were observed. Strain 91-R6 and laboratory strain R1 showed 99.63% sequence identity in common chromosomal regions and only 38 strain-specific segments. This information resolves the previously uncertain relationship between type and laboratory strains.

## ANNOUNCEMENT

Halobacterium salinarum is a well-studied model haloarchaeon first isolated from cured cod in 1922 (1). The source of this organism was found to be salt. This original type strain was lost (2), and a neotype was assigned, *H. salinarum* isolate 91-R6 (3) (NRC 34002 = ATCC 33171 = DSM 3754), which is referred to hereafter as strain 91-R6. It was isolated in Canada from the red discoloration on a salted cow hide (3). Little experimental work has been reported for the type strain, but two laboratory strains of *H. salinarum* have been sequenced, namely, strain NRC-1 (4) and strain R1 (5). Their relationship to the type strain has previously remained uncertain.

A fresh culture of strain 91-R6 (DSM 3754^T^) was obtained from the DSMZ and inoculated directly into liquid medium, omitting any colony purification. DNA from the resulting cells was used for genome sequencing using high-coverage PacBio long-read technology (5 single-molecule real-time [SMRT] cells; 253,044 reads; average length, 5,400 bp; and 1.3 Gbp total, using kits from PacBio, including template preparation, MagBead loading, and sequencing). For assembly, we used the SMRTanalysis pipeline (RS_HGAP_assembly.2 v2.3.0, Pacific Biosciences, with default parameters) which runs HGAP (DAGCON-based hierarchical genome assembly process) in three steps (6), namely, preassembly, *de novo* assembly with the Celera assembler, and final polishing with Quiver. Despite high coverage, the assembly gave 43 contigs. A supervised genome assembly was performed using Canu v1.7 (7) for assembly and Geneious v10.2 (8) for integration and editing of contigs. Considerable population heterogeneity (transposon integrations and transposon-triggered genome inversions) was encountered, which explained the failure of the automated assembly procedure. The representative genome sequence consists of 1 chromosome (2,178,608 bp, 67.1% G+C content, and 400-fold coverage) and 2 large plasmids (pHSAL1, 148,406 bp and 60.6% G+C content; pHSAL2, 102,666 bp and 56.5% G+C content; 500-fold coverage for plasmids). The plasmids share a 39,230-bp duplication devoid of any sequence difference.

The chromosomes of strains 91-R6 and R1 were compared in detail by methods we previously described for strain comparisons of *Haloquadratum walsbyi* and *Photorhabdus laumondii* (9, 10). They showed very high DNA sequence similarity (99.63% sequence identity covering 84.9% of 2.17 Mb in strain 91-R6 and 92.5% of 2.00 Mb in strain R1) and complete colinearity. Only 38 strain-specific regions were identified. As the chromosomes of strains R1 and NRC-1 are nearly identical, there is also very high similarity of the chromosomes from strains 91-R6 and NRC-1. The plasmids of strains 91-R6 and R1 exhibited patches with very high interstrain similarity (107 kb, pHSAL1/pHS3; 42.5 kb, chromosome/pHS3; and 13.3 kb, pHSAL2/pHS1).

Given the close genomic similarity of the strains, the annotation of strain R1 was used as a reference for that of strain 91-R6. Strain 91-R6 codes for 2,451 regular proteins, of which 2,092 are shared with strain R1, with only a minority (73) having less than 98% protein sequence identity. Strain R1 is 1 of 12 haloarchaeal genomes which have been reliably annotated by our gold standard protein-based strategy (5, 11). Our efforts also include regular systematic correlation with high-level databases (Swiss-Prot and KEGG).

During this project, we revised the annotation of the *H. salinarum* NRC-1 genome and submitted it to NCBI as a third-party annotation (NCBI:TPA).

### Data availability.

The genome sequence of strain 91-R6 has been deposited in GenBank under the accession numbers CP038631 (chromosome), CP038632 (pHSAL1), and CP038633 (pHSAL2). Raw reads have been deposited in the SRA archive under BioProject accession number PRJNA530823. The third-party annotation of the NRC-1 genome has been deposited in the Third Party Annotation section of GenBank (accession numbers BK010829, BK010830, and BK010831).
